# Antioxidant and Anti-Inflammatory Properties of* Anacardium occidentale* Leaf Extract

**DOI:** 10.1155/2017/2787308

**Published:** 2017-08-17

**Authors:** Natália Cabral Souza, Juliana Medeiros de Oliveira, Maurílio da Silva Morrone, Ricardo D'Oliveira Albanus, Maria do Socorro Medeiros Amarante, Christina da Silva Camillo, Silvana Maria Zucolotto Langassner, Daniel Pens Gelain, José Cláudio Fonseca Moreira, Rodrigo Juliani Siqueira Dalmolin, Matheus Augusto de Bittencourt Pasquali

**Affiliations:** ^1^Institute of Tropical Medicine of Rio Grande do Norte, Federal University of Rio Grande do Norte, Natal, RN, Brazil; ^2^Department of Biochemistry, Federal University of Rio Grande do Sul, Porto Alegre, RS, Brazil; ^3^Department of Computational Medicine and Bioinformatics, University of Michigan, Ann Arbor, MI 48109, USA; ^4^Department of Morphology, Federal University of Rio Grande do Norte, Natal, RN, Brazil; ^5^Department of Food Engineering, Federal University of Campina Grande, Campina Grande, PB, Brazil

## Abstract

In tropical America, principally in Northeastern Brazil, the leaf extract of* Anacardium occidentale *is traditionally used for treatment of different diseases. However, chemical and biological properties and activities of* Anacardium occidentale* are poorly investigated and known. Here, we evaluated the antioxidant and anti-inflammatory activities* “in vitro”* of leaf extract from* Anacardium occidentale. *Our results show that leaf extract exhibits antioxidant activity when used to treat RAW 264.7 macrophage cells. Antioxidant effects were observed by decrease in oxidative damage in macrophage cells treated with 0.5 *µ*g/mL and 5 *µ*g/mL of leaf extract. Moreover, leaf extract reversed oxidative damage and inflammatory parameters induced in LPS-stimulated RAW 264.7 macrophage cells. Leaf extract at 0.5 *µ*g/mL and 5 *µ*g/mL was able to inhibit release of TNF-*α* and IL-1*β* in LPS-stimulated cells. Taken together, our results indicate antioxidant and anti-inflammatory effects of leaf extract from* Anacardium occidentale *and reveal the positive effects that intake of these products can mediate in biological system.

## 1. Introduction

*Anacardium occidentale* is a tropical plant that occurs principally in Northeastern Brazil [1]. Commercially, the fruit (cashew) of this tree is used by industries to produce juice and derived fibers. The fruit is composed of two parts: the fruit itself, chestnuts, and the pseudo-fruit, which is used to produce juice [2]. Recently, the use of this fruit and its derivatives as a nutritional food has increased. Characteristically, cashews present different volatile compounds that are responsible for the aroma, but can also can be involved in allergenic clinical symptoms [3, 4]. Others biological compounds such as flavonoids, carotenoids, and vitamins (C and A) are also present in the fruit. These phytochemicals compounds represent an important content which can be related to significant biological activities. Moreover, the cashew extract has been used as alternative medicines for primary health care [3, 4].

Anesthetic, bactericidal, and insecticidal properties of extracts and phytochemicals derivatives from* Anacardium occidentale* have been reported by different authors [2–9]. These effects were associated with presence of anacardic acids (AAs). AAs, which are the major compounds present in the extract, juice, and fibers derived from cashew, are also related to inhibition of cancer metastases, where these compounds are able to decrease activity of matrix metalloproteinases (MMP) [8]. Other important effect of AAs is regulating the activity of histone deacetylases (HDACs) [9]. These are responsible for modulating gene expression in cells, and also can be involved in cellular differentiation and proliferation. Another effect recently demonstrated is the modulation of NF-*κ*B activity in cells treated with extracts from* Anacardium occidentale* [7].

The anti-inflammatory properties of cashew extract, however, remain unclear. Recently, it was demonstrated that stem bark extract alters the release and gene expression of cytokines, as well as TNF-*α*, IL-1, and IL-6 [7]. These alterations were observed in neuronal and microglial cells [7]. The potential anti-inflammatory effect from* Anacardium occidentale* also was observed in different models [10–13]. Evidence that antioxidant characteristics of plant extracts are responsible for attenuated levels of reactive oxygen species (ROS) and reactive nitrogen species (RNS) in biological systems was largely reported during recent years [14–16]. ROS are involved in different mechanisms that participate in normal signalling pathways and processes such as cancer development, neurodegenerative diseases, and cardiovascular diseases [17]. ROS also exert central involvement in activation and progression of inflammatory pathways [18–21]. Although metabolic process leads naturally to production of ROS as superoxide radical anion (O_2_^−∙^), hydrogen peroxide (H_2_O_2_), hydroxyl radical (OH^*∙*^), and nitric oxide (NO), excess of these products, are involved in progression of inflammatory response [18–21].

Here in this study, we investigated the antioxidant and anti-inflammatory effects of leaf extract from* Anacardium occidentale* in an “in vitro” model of inflammation. We observed that leaf extracts present antioxidant activities. Moreover, the extract was able to mediate inhibition of proinflammatory cytokines secretion such as IL-1*β* and TNF-*α* in lipopolysaccharides- (LPS-) stimulated macrophages cells. These data provide evidence that leaf extract may exert an antioxidant and anti-inflammatory role due to the properties of biomolecules presents in the extract.

## 2. Materials and Methods

### 2.1. Chemicals and Cell Culture

Chemicals and culture reagents were purchased from Sigma Chemical Co. (St. Louis, MO, USA). The macrophages cell line RAW 264.7 was grown in RPMI-1640, 10% FBS, and maintained at 37°C in an atmosphere containing 5% CO_2_. The media were supplemented with 1% penicillin/streptomycin.

### 2.2. Extract

The leaves of* Anacardium occidentale* were collected in Parnamirim, Rio Grande do Norte, Brazil. The plant material was identified by Pharmacy Faculty Center of Federal University of Rio Grande do Norte, UFRN, Brazil. Specimen from* Anacardium occidentale* was deposited at the Herbario do Departamento de Botânica, Ecologia e Zoologia of Federal University of Rio Grande do Norte, UFRN, Brazil. To prepare extract, the leaves of* Anacardium occidentale* (plant solvent 1 : 10, w/v) were air-dried at 40°C, powdered, and extracted by infusion with ethanol/water (40 : 60, v/v) for seven days. Afterwards, the extract was filtered, lyophilized, and stored at −80°C until tested.

### 2.3. Treatments

Extract was dissolved in medium. Concentrated stocks were prepared immediately before experiments by dissolved extract into medium. The solution was kept protected from light to keep extract stable. Cells were treated with different concentrations of extract (0.5 *µ*g/mL, 5 *µ*g/mL, and 500 *µ*g/mL). All treatments were initiated by adding concentrated solutions to reach final concentrations in the well.

### 2.4. Thiobarbituric Acid-Reactive Species (TBARS), Protein Carbonyls, and Protein Thiol Content

We measured the formation of TBARS during an acid-heating reaction, which is widely adopted for measurement of lipid peroxidation, as previously described [22]. Results are expressed as nmol of TBARS/mg of protein [23]. Protein carbonyls were determined based on the reaction with dinitrophenylhydrazine, as previously described [24]. Results are expressed as nmol of carbonyl/mg of protein. For protein thiol content the samples were evaluated to estimate oxidative alterations in protein thiol content, as previously described [25]. Results are expressed as mmol of SH/mg of protein.

### 2.5. Estimation of Antioxidant Enzyme Activities

Catalase (EC 1.11.1.6) (CAT) activity was determined through measuring the rate of decrease in H_2_O_2_ absorbance in a spectrophotometer at 240 nm, and the results are expressed as units of CAT/mg of protein [26]. Superoxide dismutase (EC 1.15.1.1) (SOD) activity was assessed by quantifying the inhibition of superoxide-dependent adrenaline autooxidation in a spectrophotometer at 480 nm, as previously described, and the results are expressed a units of SOD/mg of protein [18].

### 2.6. Determination of Intracellular RS Production (Real-Time Dichlorofluorescein Oxidation Assay)

Intracellular reactive species production was determined by the DCFH-DA-based real-time assay using intact RAW 264.7 cells [19].

### 2.7. MTT Assay and Sulphorhodamine B (SRB) Assay

After 24 h of leaf extract treatments, RAW 264.7 cells viability was assessed by the MTT assay [20]. This colorimetric assay was performed to assess growth of the cells. It estimates cell numbers indirectly by staining total cellular protein with SRB [21].

### 2.8. Indirect ELISA

TNF-*α* was quantified by indirect ELISA. IL-1*β* was detected by Abcam IL-1 beta Mouse ELISA (Enzyme-Linked Immunosorbent Assay) (Catalog number: ab100704) Immunoassay Kit following the manufacturer instructions.

### 2.9. Statistical Analysis

Results are expressed as mean values ± standard error of the mean (SEM); *p* values were considered significant when *p* < 0.05. Differences in experimental groups were determined by one-way ANOVA followed by the post hoc Tukey's test whenever necessary.

## 3. Results

**Figure 1:**
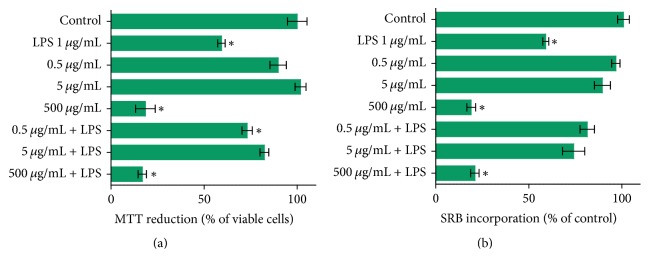
Parameters of cell viability and oxidative stress in RAW 264.7 cells treated with* Anacardium occidentale* leaf extract for 24 hours. RAW 264.7 cells were treated with leaf extract at different concentrations, 0.5, 5, and 500 *µ*g/mL. Different assays were performed to evaluate cell viability after incubation; lipopolysaccharides LPS (1 *µ*g/mL) was used as a positive control for loss of viability. (a) MTT assay and (b) SRB–incorporation assay. Control group is represented in all graphs by “Control.” Data represent mean ± SEM from three independent experiments (*n* = 6 per group). One-way ANOVA followed by the post hoc Tukey's test, ^*∗*^*p* < 0.05 versus the control group.

RAW 264.7 macrophage cells were treated with* Anacardium occidentale* extract at concentrations of 0.5 *μ*g/mL, 5 *μ*g/mL, and 500 *μ*g/mL (Figure 1) for determination of viability. Treatment with 500 *μ*g/mL of leaf extract significantly decreased cell viability during the first 24 hours. In counterpoint, this was not visualized at the two lowest concentrations as indicated through MTT and SRB assays. At the same time, we analyzed the effects of cotreatment during the inflammatory response mediated by treatment with LPS at 1 *μ*g/mL. At concentrations of 0.5 *μ*g/mL and 5 *μ*g/mL, cotreatment with the extract inhibited the decrease in LPS-mediated cell viability (Figure 1).

The levels of lipid peroxidation and carbonylation of proteins decreased at concentrations of 0.5 *μ*g/mL and 5 *μ*g/mL (Table 1) when compared to the control group in cells treated with leaf extract for 24 hours. Additionally, the cotreatment of cells with the extract blocked the LPS-mediated effect on macrophage cells in both lipid peroxidation and protein carbonylation (Table 1). In contrast, the protein thiol content was not altered by the treatments with the extract (Table 1). Interestingly, cotreatment with doses of 0.5 *μ*g/mL and 5 *μ*g/mL inhibited the effect mediated by LPS on the protein thiol content in macrophages cells (Table 1).

The activity of the antioxidant enzymes was modified during treatment with* Anacardium occidentale* leaf extract. However, these effects were only observed on LPS-stimulated cells. The activities of CAT and SOD in cells treated with leaf extract without LPS stimulation did not show significant changes (Table 1). However, it was found that leaf extract prevented the LPS-stimulated effects on CAT and SOD activities (Table 1). These effects were observed in cells treated with leaf extract at 5 *μ*g/mL. Thus, these data provide evidence that the leaf extract of* Anacardium occidentale* exhibits antioxidant properties that resulted in the inhibition of redox damage induced by LPS.

**Figure 2:**
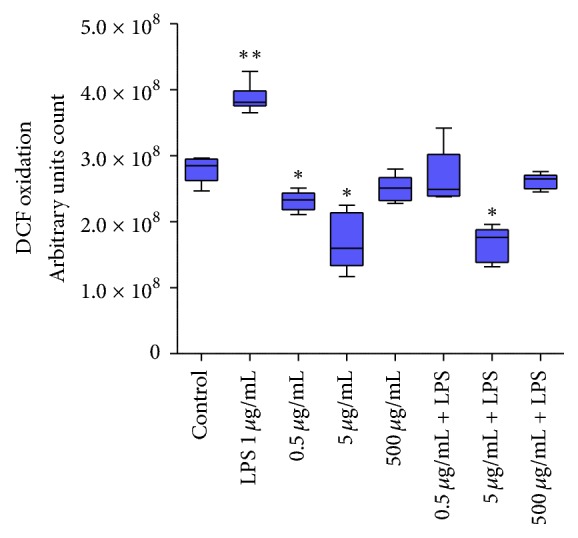
Cells were treated with different concentrations of leaf extract for 1 hour and the total production of reactive species by living cells was evaluated by the real-time DCFH oxidation assay; LPS (1 *µ*g/mL) was used as a positive control for reactive species production and fluorescence intensity was calculated relative to control cells. Control group is represented in all graphs by “Control.” Data represent mean ± SEM from three independent experiments (*n* = 6 per group). One-way ANOVA followed by the post hoc Tukey's test, ^*∗*^*p* < 0.05 versus the control group; ^*∗∗*^*p* < 0.05 versus treated group.

The real-time DCFH oxidation assay was used to evaluate the ROS/RNS production in macrophages cells treated with the* Anacardium occidentale* leaf extract. It observed inhibition in cellular ROS/RNS production at concentrations of 0.5 *μ*g/mL and 5 *μ*g/mL (Figure 2). Reaffirming the antioxidant properties of leaf extract, the production of intracellular reactive species in the cotreatment of LPS-stimulated macrophages was assessed by DCF fluorescence (Figure 2). Our results showed that the treatment with leaf extract was able to decrease the detection of ROS/RNS in DCFH assays.

**Figure 3:**
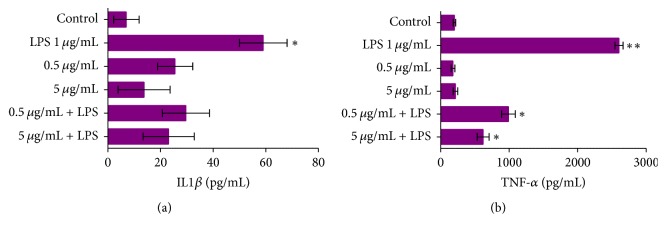
The levels of IL-1*β* (a) and TNF-*α* (b) in medium of incubation were measured. Detection of cytokines was performed by ELISA assays. Control group is represented in all graphs by “Control.” Data represent mean ± SEM from three independent experiments (*n* = 6 per group). One-way ANOVA followed by the post hoc Tukey's test, ^*∗*^*p* < 0.05 versus the control group; ^*∗∗*^*p* < 0.05 versus treated group.

Secretion of inflammatory cytokines such as TNF-*α* and IL-1*β* is induced when macrophage cells are LPS-stimulated. Here, we showed that the cotreatment with the extract of the leaves of* Anacardium occidentale *mediated a reduction in the secretion of TNF-*α* and IL-1*β* (Figure 3) at different concentrations. Taken together, these data suggest that* Anacardium occidentale* leaf extract has anti-inflammatory properties.

## 4. Discussion

The diversity of secondary metabolites produced for the plants has been extensively studied in last years, principally due to the redox, anti-inflammatory, and antitumoral properties [27–30]. In this terms,* Anacardium occidentale* is one of the species that in Northeastern Brazil have higher importance to public health [1]. Usually, the extract of this plant is used therapeutically with medicinal objectives for treatment of different diseases, including inflammation, diabetes, arteriosclerosis, respiratory diseases, and neurodegenerative disorders [1]. However, the effects presented by extracts from different tissues of the* Anacardium occidentale* remain to be elucidated. Here, our data reveal evidence that* Anacardium occidentale *leaf extract can exhibit antioxidant and anti-inflammatory activities in a biological system.

First, we investigate the viability of cells treated with* Anacardium occidentale *leaf extract. The MTT and SRB assays were carried out to evaluate the viability/toxicity of extract in RAW 264.7 macrophage cells. The higher dose of 500 *μ*g/mL of extract treatment induced significantly the decrease in cell viability. In the lowest concentration the* Anacardium occidentale *leaf extract did not show alterations in cell viability. When the cells were LPS-stimulated, the lowest doses of 0.5 *μ*g/mL and 5 *μ*g/mL cotreatment inhibited the decrease in cell viability mediated by LPS. It is known that phytochemical composition of the plant* Anacardium occidentale* presents phenolic compounds, such as quercetin, anthocyanins, and tannins [31–33]. These phytochemical compounds are related to several biological activities, including antioxidants, apoptotic, antiapoptotic, and anti-inflammatory activities [34]. Previous studies have demonstrated that treatment with LPS in macrophages cells leads to impairment of the electron transfer system, thus increasing the rate of O_2_^−∙^ production, and, respectively, the damage in lipids, proteins, and DNA [35]. The consequence of this damage is a decrease in cell viability. Our results showed that leaf extract cotreatment inhibited the decrease in cell viability of LPS-stimulated cells. We suggest that the effect observed in cell viability can be associated with different compounds present in* Anacardium occidentale* leaf extracts, which are associated with antioxidant properties.

It is known that production of ROS/RNS is a process that occurs naturally during cellular metabolism; however higher levels of ROS/RNS production have been associated with impairment of metabolic functions [36]. Plants and derivatives present a diversity of antioxidant and anti-inflammatory properties. Based on these characteristics, there is a stimulation to the intake of these products, principally due to it helping in maintaining the functions in biological systems [30]. Additionally, several studies have demonstrated the involvement of ROS/RNS with increased risk and incidence of chronic diseases such as arteriosclerosis, respiratory diseases, diabetes, and neurodegenerative disorders [37]. These findings reinforce the use of plant extracts through tea, juice, or food supplementation as a mechanism to improve the treatment and/or prevention of those different pathological conditions [38, 39]. Here, our results showed for the first time that* Anacardium occidentale* leaf extract treatment at different doses exhibits antioxidant and anti-inflammatory effects in macrophage cells.

The extract inhibited the effects mediated by LPS on redox parameters when used as cotreatment in macrophage cells that were LPS-stimulated. Leaf extract decreased lipid peroxidation, protein carbonylation, and protein thiol content. The activities of CAT and SOD were also modulated by leaf extract of* Anacardium occidentale*. A class of compounds present in* Anacardium occidentale* that have been associated with antioxidant, anti-inflammatory, and antitumoral effects are AAs [8, 33, 40–45]. These compounds likely contribute to reduce ROS/RNS. Furthermore, AAs are effective in inducing inhibition of prooxidant enzymes responsible for ROS/RNS production. AAs also present chemical properties of chelate divalent metal ions and therefore can reduce reactions that trigger ROS/RNS production, such as Fenton reaction [40, 46, 47]. In LPS-stimulated cells the formation of both O_2_^−∙^ and hydroxyl radical (OH^*∙*^) is related to oxidative damage observed in cells. Our results in ROS/RNS production, evaluated through DCF assay, provide evidence that LPS induces these alterations. Interestingly, the cotreatment with leaf extract prevents these alterations. Taken together, our data suggest that* Anacardium occidentale* leaf extract presents antioxidant properties that induce the cellular response to injury, principally in redox parameters.

During proinflammatory response in LPS-stimulated cells the release of cytokines such as IL-1*β* and TNF-*α* occurs characteristically. IL-1*β* action is well characterized in terms of IL-1 cytokine family [48]. The blocked IL-1*β* action/secretion have been associated with anti-inflammatory responses [48]. Our results demonstrated that cotreatment with leaf extract at different doses was able to mediate the inhibition of IL-1*β* secretion in macrophages that were LPS-stimulated. The importance of inhibition of IL-1*β* secretion/activation has been associated with decrease in inflammasome activation, and therefore the physiological response mediated principally for macrophage cells [18–20]. TNF-*α* secretion also corroborates the immunological system for inducing inflammation in pathological conditions [49]. In target cells TNF-*α* is involved in ROS/RNS production [50]. It is yet unclear the mechanisms involved TNF-*α* pathway signalling for inducing ROS/RNS production; however it has been demonstrated that this cytokine alters mitochondrial function and as a consequence increases ROS production [51]. Here, we showed that* Anacardium occidentale* leaf extract treatment blocked secretion of cytokines such as IL-1*β* and TNF-*α* in macrophage LPS-stimulated cells. These findings demonstrate that this extract can present anti-inflammatory properties. Our data suggest that both anti-inflammatory and antioxidant effects observed in our study are derivatives from synergistic effect of biological compounds present in leaf extract of* Anacardium occidentale*.

In conclusion, the results presented here demonstrate for the first time that the leaf extract of* Anacardium occidentale* exhibits antioxidant and anti-inflammatory properties in a biological system. Moreover, our data provide evidence that reinforces the importance of potential health benefits that the consumption of* Anacardium occidentale* derivatives may promote. Our findings also contribute to improving the comprehension of the properties and mechanism of action mediated by* Anacardium occidentale* biological compounds.

## Figures and Tables

**Table 1:** Redox parameters, TNF-*α* and IL1*β* levels in macrophage cells, RAW 264.7.

			*Anacardium occidentale* leaf extract						
			Extract				Extract + LPS 1 *μ*g/mL		
	Control	LPS 1 *μ*g/mL	0.5 *μ*g/mL	5 *μ*g/mL	500 *μ*g/mL		0.5 *μ*g/mL	5 *μ*g/mL	500 *μ*g/mL
Carbonyl groups (nmol/mg prot)	6.38 ± 0.47	10.02 ± 0.44^*∗∗*^	4.13 ± 0.17^*∗*^	3.97 ± 0.19^*∗*^	2.54 ± 0.16^*∗*^		6.22 ± 0.28	7.65 ± 0.55	2.02 ± 0.25^*∗*^
TBARS (nmol/mg prot)	0.61 ± 0.03	0.81 ± 0.05^*∗∗*^	0.45 ± 0.01	0.30 ± 0.02^*∗*^	0.21 ± 0.01^*∗*^		0.55 ± 0.04	0.45 ± 0.03	0.12 ± 0.01^*∗*^
Thiol groups (*μ*mol/mg prot)	143.81 ± 5.25	72.75 ± 1.95^*∗∗*^	184.42 ± 5.70	153.88 ± 3.60	63.52 ± 3.32^*∗*^		122.05 ± 5.59	140.33 ± 4.60	49.90 ± 1.97^*∗*^
SOD (U/mg prot)	11.64 ± 0.16	6.03 ± 0.13^*∗∗*^	11.89 ± 0.18	12.27 ± 0.34	5.61 ± 0.20^*∗*^		10.46 ± 0.28	9.29 ± 0.33	3.71 ± 0.18^*∗*^
CAT (U/mg prot)	0.90 ± 0.04	0.45 ± 0.03^*∗∗*^	0.94 ± 0.06	0.78 ± 0.02	0.29 ± 0.02^*∗*^		0.78 ± 0.03^*∗*^	0.85 ± 0.04	0.30 ± 0.05^*∗*^

Parameters of oxidative stress: carbonyl levels were quantified in order to evaluate cell protein oxidative damage; thiobarbituric acid reactive species (TBARS) levels were assessed as an index for cellular lipid peroxidation; and thiol levels were assessed to verify protein redox modification. The activities of the antioxidant enzymes catalase (CAT) and superoxide dismutase (SOD) were also evaluated. Data represent mean ± SEM from three independent experiments (*n* = 6 per group). One-way ANOVA followed by the post hoc Tukey's test, ^*∗*^*p* < 0.05 versus the control group; ^*∗∗*^*p* < 0.05 versus treated group.
